# Adding Flavours: Use of and Attitudes towards Sauces and Seasonings in a Sample of Community-Dwelling UK Older Adults

**DOI:** 10.3390/foods10112828

**Published:** 2021-11-17

**Authors:** Annie Thomas, Charlotte Boobyer, Zara Borgonha, Emmy van den Heuvel, Katherine M Appleton

**Affiliations:** Research Centre for Behaviour Change, Department of Psychology, Faculty of Science and Technology, Bournemouth University, Poole BH12 5BB, UK; s5073059@bournemouth.ac.uk (A.T.); i7696550@bournemouth.ac.uk (C.B.); i7258579@bournemouth.ac.uk (Z.B.); mvandenheuvel@bournemouth.ac.uk (E.v.d.H.)

**Keywords:** taste, flavour, food intake, health, natural flavours, individual differences

## Abstract

Adding flavours can encourage food intake in older adults for health benefits. The use and attitudes of 22 community-dwelling UK older adults (15 females, aged 65–83 years) towards foods and products that add flavour, e.g., sauces and seasonings, were investigated. Participants used foods/products to add flavour when cooking and eating from 0 to 17 times/day. Taste and flavour were important, and foods/products could add flavour, make foods more pleasant and did not cause discomfort. There were concerns, however, over the healthiness of some foods/products, while consuming a healthy diet and one’s health were important. Reasons for adding flavours largely centred around ‘meal enhancement’, reasons for not adding flavours focused on ‘the product itself’ and ‘characteristics of the meal’, but there was ‘variation’ and many ‘individual differences’. Our findings highlight the benefits of adding flavours for food intakes, particularly the use of naturally flavoursome foods, such as herbs, spices, onion and garlic.

## 1. Introduction

Many older adults do not consume sufficient foods to meet their needs, resulting in undernutrition or high malnutrition risk [1,2,3]. Low intakes and undernutrition are associated with adverse health consequences including decreased muscle mass and function; decreased bone mineral density and mass; decreased strength, mobility, independence and quality of life; and increased risk of falls, fractures, frailty and mortality (e.g., [2,4,5,6,7,8,9,10,11,12]). Increased intakes, furthermore, have been found to reverse or ameliorate these issues [2,4,7,8,12,13,14].

Many reasons have been provided for low food consumption in older adults, including poor appetite; sensory deterioration; poor gastric mobility and function; poor physical abilities such as reduced dentition, poor manual dexterity and physical disability; and social concerns such as bereavement, loneliness, and reduced finances [15,16,17,18,19,20,21,22,23,24,25,26,27]. Key reasons that are repeatedly provided by older adults themselves relate to taste and enjoyment: that foods are unpleasant, bland or have uninteresting flavours. Qualitative studies report liking, taste and flavour as highly important for older adults [16,17,19,25], and questionnaire studies report liking and taste as key predictors of food intake [15,22,26,28,29]. 

Flavour enhancement (an increase in the intensity of an existing flavour) and additional flavours (an increase in the number of flavours) can directly increase food intakes. Flavour enhancement resulted in the increased consumption of flavour-enhanced compared to non-enhanced food items in residential care homes [30]. Flavour enhancement, plus the addition of flavours using monosodium glutamate (MSG) also increased food consumption in hospitalised patients [31], increased dietary intake and body weight in nursing home residents [32] and increased food satisfaction in healthy older adults [31]. Added natural flavours such as ginger, garlic and sesame oil increased intakes in older hospitalised patients [33], and flavours such as lemon, garlic, salt and pepper increased intakes in individuals with Alzheimer’s disease [34]. Pouyet et al. [35] and Kimura et al. [36] also demonstrated increased intakes following the addition of sauces to foods in individuals with Alzheimer’s disease, and we have demonstrated increased energy and protein intakes following the addition of sauces and seasonings to meals for care home residents and community-dwelling older adults [37,38,39]. We recently also demonstrated increased egg intakes in community-dwelling older adults following the provision of recipes designed to increase taste, flavour and variety [40]. 

Many older adults report reduced sensory abilities, including reductions in taste sensitivity and olfactory discrimination [24]. Flavour enhancements are considered to compensate for these reduced acuities [23,24,32,41], to result in increased taste, food palatability and salivary flow, easing food consumption and digestion [24,32]. Added flavours can also increase other sensory aspects of a food or meal, such as texture and lubricity [34,36,37,39,42], and may add variety to an eating experience [36,40,43,44]. Improvements in a range of sensory aspects, such as taste, aroma and texture, have been found to increase food intakes in older adults [42,44]. Many flavoursome foods, such as herbs and spices, furthermore, may have health benefits of their own [45,46,47,48,49], although evidence for clinically relevant effects in humans is limited [45,49], and effect sizes will be further reduced by the quantities typically consumed in a meal. The use of these foods to replace and reduce less desirable ingredients, such as salt, and to promote healthy food consumption has been recommended [41,42,49].

Not all studies, however, report clear increases in food preferences or consumption as a result of flavour enhancement or addition. Griep et al. [50] reported greater preferences for three flavour-enhanced products among older adults but greater intakes for only one. Essed et al. [51] report no changes to intakes following flavour enhancement or added flavours for sixteen weeks, and a recent review suggests limited impacts for flavour enhancement on food preferences and intakes [18]. Some individual differences, however, have also been reported. Essed et al. [51] and Schiffman and Warwick [30] report increased intakes in those with poorer olfactory and/or gustatory abilities, and Kremer et al. [42] found increased liking for some flavour- and sensory-enhanced food products but for not others, depending on olfactory ability. Other individual differences have also been reported [34,38,52].

Wide variation in eating behaviour in older adults may be unsurprising given the range of age-related changes in physiological and social concerns [18,23,24,27]. This variation suggests that recommendations to enhance or add flavours to foods to aid intakes for all older adults may be rather basic. The addition of flavours may offer a straightforward, practical recommendation that aids the health of many older adults, but very little work investigates the attitudes and actions of older adults themselves. While efforts to increase intakes tend to focus on those in residential care, higher food intakes for community-dwelling older adults will also be of value; yet, for community-dwelling older adults, attitudes towards adding flavours will be fundamental to the success of any related recommendation. This study aimed to explore the use of and attitudes towards sauces and seasonings in a sample of community-dwelling UK older adults. 

## 2. Materials and Methods

### 2.1. Participants

Older adults were recruited from the local community in and around Bournemouth, UK, via newsletters and personal contacts, for an interview study on ‘Tastes and flavours as we age’. Participants were required to be aged 65 years or over and living in their own homes, such that food intake was largely under their own control. No other inclusion criteria were used so as to include a sample that was as generalizable as possible. 

### 2.2. Data Collection

Participants completed two questionnaires and an interview on their use of and attitudes towards sauces, seasonings and other flavoursome food items.

#### 2.2.1. Questionnaire on Use and Attitudes

Participants were first asked how often they used each of the following food items: (1) ‘when cooking’: ‘salt and/or pepper’; ‘herbs and/ or spices’; ‘onion and/or garlic’; ‘seasoned/flavoured butters and/or oils’; ‘stock cubes, wine, other seasonings’; ‘specific recipes’; ‘other items to specifically add taste’; and (2) ‘when eating’: ‘salt and/or pepper’; ‘herbs and/or spices’; ‘onion and/or garlic’; ‘seasoned/flavoured butters and/or oils’; ‘table sauces, e.g., mustard, gravy’; ‘recipe or packet sauces, e.g., parsley sauce’; ‘meat-specific sauces, e.g., mint sauce, apple sauce’; ‘pickles, chutneys’; ‘other’. Response options were ‘every meal’, ‘once a day’, ‘3–4 times a week’, ‘once a week’, ‘once a fortnight’, ‘once a month’, ‘rarely/never’, scored to provide a measure of use per day. A range of sauces, seasonings and flavoursome foods were included, to allow consideration of a number of routes through which flavours could be added to foods. Second, participants were asked how much they agreed or disagreed with 13 attitudinal statements about sauces and seasonings, e.g., ‘Sauces and seasonings make foods more pleasant’; and 13 attitudinal statements on diet and health, e.g., ‘I try to have a healthy diet’. Response options were ‘strongly disagree’, ‘disagree’, ‘neither agree not disagree’, ‘agree’ and ‘strongly agree’, scored −2 to +2 respectively. Statements were based on previous work assessing attitudes towards healthy eating and food choice [53,54].

#### 2.2.2. Interview

Participants were then interviewed on the following topics: why they used/did not use sauces, seasonings or other foods to add flavour; whether they used other strategies to flavour foods; whether they did anything else to deliberately enhance, change or mask the flavours of foods or to make foods tastier or more pleasant. Following these questions, participants were given a brief (220-word) magazine article on three studies demonstrating increased consumption in older adults following the addition of sauces and seasonings to meals (see Supplementary Materials). Participants were then asked whether they may think differently about flavouring food in future; whether they would be willing to try some sauces or seasonings or new recipes; and of any barriers that may arise to these activities. All participants were asked the same questions following an interview schedule. 

#### 2.2.3. Questionnaire on Future Use and Attitudes

A second questionnaire then asked for agreement or disagreement to: 2 questions on future intentions to use sauces and seasonings, 3 questions on abilities towards using sauces and seasonings, 10 of the attitudinal statements in the first questionnaire reworded as if for the future, 3 questions on likely impacts on diet and health, and 2 questions on adverse effects. Response options were ‘strongly disagree’, ‘disagree’, ‘neither agree not disagree’, ‘agree’ and ‘strongly agree’, scored −2 to +2, respectively.

#### 2.2.4. Demographic Details

Details were also collected on gender, age, highest educational award, employment prior to retirement, number of holidays in the last 12 months as a measure of affluence, denture-wearing and existence of a medical condition or taking medication that may influence taste or flavour perception.

Questionnaires and interviews were administered by one of two female young adult researchers (C.B. or Z.B.) at Bournemouth University, UK, from June to July 2017. All interviews were recorded and subsequently transcribed. Participants were offered a range of complimentary sauces and seasonings following participation as a token of thanks.

### 2.3. Data Analyses

Questionnaire data were analysed using descriptive statistics to describe the sample and their sauce and seasoning use. Attitudes were investigated using one-sample t-tests in the whole population, to investigate a difference from the mid-point of the scale. Differences in attitudes between low, medium and high users of sauces and seasonings (defined using tertiles) were also explored using one-way ANOVA. Significance was considered at *p* ≤ 0.01, to account for the number of statistical tests undertaken. 

Interview data were transcribed by the two interviewers (C.B. and Z.B.) and analysed by thematic analysis using an inductive approach. Analyses were based on the six steps of analysis by Braun and Clarke [55] by both interviewers (C.B. and Z.B.) and repeated and confirmed by a third independent analyst (A.T.).

## 3. Results

### 3.1. Participants

In total, 22 volunteers took part: 15 females, 7 males, mean age = 72.3 (SD = 5.2), range 65–83 years. The majority of the sample had a college or university education (*N* = 12, 55% participants) or a professional education beyond university (*N* = 5, 23% participants) and reported themselves as in professional and managerial employment prior to retirement (*N* = 18, 82% participants). Seven (32%) participants had been on holiday more than twice in the past 12 months, six (27%) participants had been on holiday twice, two (9%) participants had been on holiday once and seven (32%) participants had not been on holiday at all. One participant wore full dentures, five participants wore partial dentures and one participant had a medical condition and took medication that they felt affected taste/flavour perception. 

### 3.2. Use of Sauces and Seasonings

The frequency of adding flavours during cooking and eating is given in Table 1. The majority of participants reported using flavours more than once per day for cooking *(N* = 15, 68%), while nine (41%) participants reported very high use (more than three times per day) and two (9%) participants reported minimal use (less than once per week). The majority of participants reported using flavours more than once per day during eating (*N* = 12, 55%), while four (18%) participants reported very high use (more than three times per day) and three (14%) participants reported minimal use (less than once per week). When use for cooking and eating were combined, six (27%) participants added flavours to foods more than six times per day (up to 17 times) (high users), nine (41%) participants added flavours to foods 1.5–5.5 times per day (moderate users), and seven (32%) participants used flavours less than 1.5 times per day (low users).

### 3.3. Attitudes towards Sauces and Seasonings

Attitudes towards adding flavours in the sample as a whole *(N* = 22) are given in Table 2. Sauces and seasonings were considered to add flavour to foods, make foods more pleasant and not to cause discomfort. However, the sample also preferred to add their own sauces and seasonings to foods because sometimes there can be too much, and sauces and seasonings were not considered to contribute to a healthy diet. It was important to the sample that their foods were pleasant, tasty and healthy, and health was important. No differences were found between high (*N* = 7), moderate (*N* = 9) and low (*N* = 6) users. Data from the different users are given in the Supplementary Materials.

### 3.4. Reasons for Adding and Not Adding Flavours to Foods

Five themes emerged from the qualitative analyses: ‘meal enhancement’, ‘the product itself’, ‘characteristics of the meal’, ‘variation’ and ‘individual differences’. Details of the themes and subthemes are provided in Table 3, with some illustrative quotes. Further quotes are also provided in the Supplementary Materials. Reasons for using sauces and seasonings largely centred around ‘meal enhancement’, reasons for not using sauces and seasonings focused on ‘the product itself’ and ‘characteristics of the meal’. A lot of ‘variation’, however, was also found where sauces were considered appropriate on some occasions and in some situations, and not in others, largely dependent on context. ‘Individual differences’ were also found based on personal preferences, personal experiences, ethical choices, health beliefs, personal circumstances and age-related concerns. No clear patterns were seen in the interviews or the emerging themes between those who were high, moderate or low users.

### 3.5. Attitudes towards Future Use

Attitudes towards sauce and seasoning use in the future are given in Table 4. Participants were confident that they could use sauces and seasonings in the future if they wished and that this would not cause discomfort. The interview also did not have adverse consequences.

## 4. Discussion

Several findings emerge from this study. Firstly, taste, flavour and pleasure were important aspects of food consumption for older adults, and sauces and seasonings were recognised to add flavours and pleasure. Secondly, looking after one’s health and consuming a healthy diet were also important, but sauces and seasonings were considered not to contribute to a healthy diet. Thirdly, sauce and seasoning use was very varied within the sample, and a recognition of individual differences was considered key.

In relation to taste, flavour and pleasure, our sample agreed that it was important to them to consume foods that were tasty and pleasant and that sauces and seasonings could add flavours to foods and make foods more pleasant. Reasons for using sauces and seasonings largely centred around ‘meal enhancement’: sauces and seasonings could enhance, increase or improve the taste, smell and/or flavours of a meal; could result in variety and improved texture or digestibility; and could result in enhanced enjoyment from the act of cooking as well as eating. Preferences for taste and flavour have previously been found repeatedly in older adults, as has the use of flavours to enhance the pleasure gained from foods and eating [24,38,43,50,56]. The value of added flavours for meal enhancement may, furthermore, be high for older individuals, due to the chemosensory losses that can affect this population [18,23,24]. The addition of flavours will also increase the variety of flavours in an eating experience, possibly also increasing food intake [34,36,43,44]. Some researchers have suggested that this increased variety may be more important to food intake than the addition of flavour *per se* [44]—a suggestion that may explain the absence of benefits in studies that employ only flavour enhancement. Some flavoursome items have also been reported to change the texture and improve the digestibility of foods, and may overcome adverse effects related to chewing and swallowing difficulties [21,35,36,37,39].

In relation to health, our sample agreed that looking after one’s health and consuming a healthy diet were important. A role for healthiness in food choice has been demonstrated previously in older populations [15,16,17,28,42,57,58,59], and this may be of particular importance for older compared to younger adults, as health may be more of a concern [16,58,60]. Some studies, however, also suggest that health can be of reduced importance, compared to taste, for some older individuals [17,19,22].

Interestingly, however, sauces and seasonings were considered not to contribute to a healthy diet. Some qualitative comments also suggested concerns or suspicions over ‘the product itself’; concerns about product ingredients, commercial products, shelf-life and brand reputation. Preferences were demonstrated for natural foods or natural food components, and other studies also suggest this preference, plus a reduced preference for artificial products by older consumers [56,58,59]. Previous work suggests that older adults can conceptualise healthy eating to be a result of fresh, natural ingredients and tend to display a dislike of convenience foods [61], suggesting that ready-made sauces may be perceived as unhealthy. Some participants in our study did indicate a preference for homemade over commercial sauces or preferred a meal without additions. 

The idea that commercial sauces and ready meals are unhealthy can also be found in the literature, where these food items are considered to add salt and fat to the diet [20,62,63]. The reality of the health detriments compared to the health benefits, however, may be low. In our studies, adults typically consumed 8 g seasoning, providing an additional 1 g salt (16% guideline daily allowance) [39] or 32–56 g sauce, providing an additional 0.8–1.2 g or 33–45 kJ energy from fat and 0.4–0.6 g salt (7–10% guideline daily allowance) [37,39]. Care must be taken, but these values compare to typical increases of 72–176 kJ energy and 25–100 kJ energy from protein from meals with sauces and seasonings compared to those without [37,39]. In the study by Mathey et al. [32], added MSG contributed 30–45 mg sodium/day (0.3–0.5% average sodium intakes) and resulted in mean daily energy intakes of an additional 277 kJ. Added benefit may also be gained from flavoursome foods with additional health benefits, such as protein-rich cheese- and dairy-based sauces [64] or from seasonings or sauces that have been fortified [65]. Perceptions that sauces and seasonings are unhealthy are important. Many individuals will not consume foods that they consider to be detrimental to their health (without an alternative benefit), or if this is requested will consume less than they may otherwise. Perceptions that sauces and seasonings are unhealthy, thus, may not only explain findings in studies where added flavours do not increase intakes, but may also sabotage any attempts to increase intakes through promoting the addition of flavours.

In relation to individual differences, sauce and seasoning use was varied. The sample agreed that they preferred to add their own sauces and seasonings, allowing choice over quantity, and many qualitative comments highlighted variation in sauce use. This variation was found, both within and between participants, where some sauces and seasonings were considered only appropriate in certain environments, on certain occasions or for certain meals. Certain flavours were also added more or less often dependent on local availability, sometimes based on location or climate. These findings suggest that added flavours can enhance or improve the flavour of a meal but that only certain added flavours may be acceptable for certain meals or in certain circumstances. Many cooking recommendations, including many commercial seasoning products, recognise the common use of specific flavour combinations or the matching of flavours with particular food items [49,66,67,68]. These pairings are largely based on flavour principles, and acceptability to the consumer is strongly influenced by culture and familiarity [49,66,67,68]. Variation in preferred flavours and flavour intensities is unsurprising [66,67,68], as is variation based on meal type, occasion and dining environment [66,67].

Strong individual differences were also found based on ‘personal preferences’, ‘personal experience’, ‘ethical choices’, ‘health beliefs’, ‘personal circumstances’ and ‘age-related concerns’. These individual differences are found for the consumption of almost all food items in all populations, and individual differences in food choice among older adults are frequently reported [52,56,69], but influences related to experience, circumstance and age-related concerns are often magnified for older adults [15,16,17,18,19,22,23,27,61].

## 5. Implications

To take this work forward, our findings suggest a need for increased awareness of the benefits of adding flavours to foods to increase food intakes that may be of health benefit to older adults, but we also demonstrate a need for sensitivity to existing perceptions towards adding flavours and flavoursome foods. Our findings suggest, firstly, that a focus on taste and enjoyment may be useful. Recommendations that aim to enhance taste and enjoyment have previously been offered [16,23], to include the development of ‘foods with richer tastes and strong but appetizing smells’ [16,23] and the use of products providing a variety of sensory characteristics [23,34,42]. 

Given the concerns over commercial products, however, an increased focus on the use of foods that are naturally flavoursome such as herbs, spices, lemon, onion and garlic may have greater impact. The use of natural flavours has been recommended [33,41] and focus on specific flavour pairings or combinations that enhance the natural flavours of other foods [49,66,67,68] may also be desirable. A recent study that failed to find effects of added flavours suggested explanations based on flavour combinations and appropriateness [70]. A focus on flavours that naturally enhance those of other foods may also dispel fears that adding seasonings during eating is a sign that a meal is tasteless or poor quality. Some participants mentioned a need for care when adding seasonings to avoid insulting a chef. A focus on traditional flavour pairings, such as pork with apple and turkey with cranberry in the UK, may also be helpful considering a common reluctance among older adults to try new or novel foods and dishes [59,71,72]. 

Information on the health benefits of specific flavoursome ingredients, such as spices, garlic and capsicums may also encourage consumption [45,46], although some caution regarding small effect sizes [45,49] and possible adverse events may also be needed [46,47,48]. Some participants in our study reported that certain spices, such as chilli and onion, can cause digestive discomfort, but there was also recognition that these concerns apply to limited foods and/or to limited individuals [19]. Our sample, as a whole, disagreed that sauces and seasonings cause pain or discomfort. 

Participants were also confident that they could use sauces and seasonings in the future if they wished; thus, there may be benefit to promotional strategies. Educational interventions for older adults have previously been found to be beneficial, particularly where messages are clear, simple, limited in number and tailored or relevant to specific circumstances [60,61,73], but limited impacts are also reported [73], where older adults can be resistant to education following a lifetime of learning for themselves [19,29,61]. Older adults as a population group can also be difficult to reach as many do not consider themselves as old or of special consideration [17,59,74]. Older adults have also been found to reject nutritional advice and nutritional aids as unsuitable for them [29,75,76], resulting in recommendations for ‘food first’ approaches and the use of familiar foods and practices for improving nutrition and health, where possible [21,32,33,37,38,39,40,42,50,72,76,77]. Encouraging taste and enjoyment for everyone through the use of natural flavours as a means of complementing existing dishes may suit all requirements. Recognition of individual differences, however, will also remain important [15,23,24,38] and may suggest greater benefit from adding flavours during eating on an individual basis as opposed to as part of a cooking or standardised serving process. Some studies demonstrate a specific advantage to allowing individuals to select flavours and seasoning foods to personal tastes [39,43,44]. 

As a result of the mixed methods approach, the study gained considerable depth on the use of and attitudes towards adding flavours to foods in community-dwelling older adults. Our sample also included a range of higher, moderate and lower users of flavours providing increased variety. The provision of the magazine article and packets of sauces also added elements to a traditional interview study, allowing increased insights, and participants did not report any adverse consequences as a result of the interview experience. The study is limited by the use of a limited sample. Our participants, in the majority, were well educated and were likely to be of a higher income than the average UK older adult, and so they may have been more likely to consume good quality foods or express concerns over food quality than individuals with lower income or lesser education. Some research also suggests that high-income groups can place a higher value on sensory appeal than low-income groups [58]. There may also have been some response biases, due to social desirability or due to an unwillingness to admit poor health or dietary habits. Our questions also combined sauces, seasonings and flavoursome foods considering our interest in flavour, but attitudes towards sauces, seasonings, and specific foods may differ, and some limited evidence of these differences was found in our data. Considering differing health implications as a result of the consumption of differing food items, investigation of attitudes towards sauces and seasonings separately and towards specific flavoursome food items may be of interest. 

## 6. Conclusions

In conclusion, our study suggests that dietary taste and flavour are important to community-dwelling older adults and that added flavours can enhance or improve the eating experience. Our study also found that looking after one’s health and consuming a healthy diet were important, but sauces and seasonings were considered not to contribute to a healthy diet. Our findings highlight the benefits of adding flavours to increase food intakes that may enhance the health of community-dwelling older adults and suggest a particular focus on food items that are naturally flavoursome, such as herbs, spices, onion and garlic. Respect for individual differences in preferences, habits and personal needs, however, will also be important. 

## Figures and Tables

**Table 1:** Mean, SD, minimum and maximum frequency (times per day) of adding flavours to foods or meals when cooking and when eating, and number of participants reporting minimum and maximum use (*N* = 22).

	Mean	SD	Min.	N (Min.)	Max.	N (Max.)
**When cooking**						
Salt and/or pepper	1.12	1.24	0	6	3	6
Herbs and/or spices	0.59	0.66	0	1	1	1
Onion and/or garlic	0.36	0.32	0	1	1	1
Seasoned/flavoured butters/oils	0.23	0.36	0	12	1	1
Stock cubes, wine, other seasonings	0.29	0.35	0	4	1	1
Specific recipes	0.34	0.65	0	6	3	1
Other items to specifically add taste	0.18	0.73	0	20	3	1
**When eating**						
Salt and/or pepper	0.66	1.03	0	11	3	3
Herbs and/or spices	0.25	0.67	0	15	3	1
Onion and/or garlic	0.12	0.26	0	16	1	1
Seasoned/flavoured butters/oils	0.1	0.29	0	18	1	1
Table sauces, e.g., mustard, gravy	0.24	0.32	0	8	1	1
Recipe or packet sauces, e.g., parsley	0.01	0.03	0	16	0.07	1
Meat-specific sauces, e.g., mint, apple	0.06	0.11	0	11	0.5	1
Pickles, chutneys	0.10	0.17	0	9	0.5	1
Other items to specifically add taste	0.03	0.13	0	17	3	1

**Table 2:** Mean, SD score from −2 to +2 for all attitudinal statements in the questionnaire on sauce and seasoning use and attitudes, in the sample as a whole (*N* = 22). Statistics (*t* values) demonstrate a difference from 0 (no opinion).

	Whole Sample (*N* = 22)			
	Mean	SD	t(21)	*p*
**SS add flavour to foods/make foods more tasty**	**1.0**	**0.8**	**6.08**	**<0.01**
**SS make foods more pleasant/likeable**	**0.6**	**0.9**	**2.95**	**<0.01**
**SS disagree with me/cause me pain**	**−1.1**	**0.7**	**7.78**	**<0.01**
I am used to using SS	0.2	1.3	0.70	0.49
SS encourage me to eat more food/make food easier to eat	−0.4	0.9	1.84	0.08
**SS encourage me to eat more healthy food/contribute to a healthy diet**	**−0.6**	**0.9**	**2.95**	**<0.01**
**I prefer to add my own SS as sometimes there can be too much**	**1**	**1.0**	**4.82**	**<0.01**
Dishes without SS do not taste pleasant	0	1.3	0	0.99
**It is important to me that the foods I eat are pleasant/enjoyable**	**1.5**	**0.5**	**13.10**	**<0.01**
**It is important to me that the foods I eat are tasty**	**1.4**	**0.5**	**13.58**	**<0.01**
It is important to me that the foods I eat are familiar to me	−0.4	1.2	1.51	0.02
**It is important to me that the foods I eat are healthy/I try to have a healthy diet**	**1.4**	**0.7**	**9.66**	**<0.01**
**My health is important to me**	**1.6**	**0.5**	**15.39**	**<0.01**
It is important to me that other people think I have a healthy diet/would not criticise my diet	−0.3	1.0	1.30	0.21
It is important to me that other people think I am healthy/not unhealthy	0	0.9	0.23	0.82

SS: sauces and seasonings. Significance set at *p* ≤ 0.01, significant effects are highlighted in bold.

**Table 3:** Themes table from the qualitative analysis of the interviews, demonstrating themes, subthemes and example quotes. Further example quotes are provided in the Supplementary Materials.

Theme	Subtheme	Example Quotes
Meal Enhancement	Improves taste: Added SS improve the taste of the meal.	P3: “I may put some herbs in it. I may add a sauce, to make them (foods) taste nicer.” P16: “Salmon hasn’t got much of a taste for me, it hasn’t, so really that deserves a sauce.”
	Enhances smell: Added SS improve the overall smell of foods.	P1: “Rosemary it smells very good.”
	Increases flavours: There are more flavours in the dish with added SS.	P3: “If you add lemon to fish, it enhances the flavour. If you add herbs to eggs, an omelette, it adds more flavours.”
	Changes in the composition/texture of foods: Added SS change the overall texture of the meal	P7: “I’ve discovered chilli sauce and I put it on chips, as it seems to counteract any grease.”
	Aiding digestion/ability to eat: Added SS can improve digestion, often due to texture and lubrication	P1: “Adding sauces to food helps it go down more easily.” P17: “It’s (sauce) a lubricant though, which is an aid to swallowing.”
	Cooking enjoyment: Enjoyment in cooking with using additional ingredients	P20: “I have parsley, sorrel, sage, rosemary, rocket, bay tree, just about everything you can mention. I really like cooking with flavourings.”
The Product Itself	Product ingredients: Importance of the specific ingredients, often with a focus on natural ingredients	P13: “I will make my own sauce to go with the meal rather than buy a bottle. Purely because what’s in the bottle, you don’t know what’s in it.”
	Product reputation: The reputation of a product influences SS usage	P12: “I do tend to buy those off the shelf. But again, certain brands that I know and that I trust.”
	Shelf-life/Longevity: Product shelf-life influences SS usage	P2: “You can’t just buy a jar because it goes off, so I don’t have it in the house.”
	Ingredient wastage: Likely product waste can influence SS usage	P17: “What might put me off, if I had to go and buy 10 pots of particular spices that I wouldn’t be using again,”
Characteristics of the Meal	Preference for natural flavourings/taste: A preference for natural tastes that can affect SS usage	P13: “If you like chicken, why spoil it?”
	Quality ingredients: Perceived quality of the meal ingredients influences SS usage	P7: “If the meat is decent then you have the flavour in it, so you shouldn’t need to mess it about with sauces.” P12: “If the meat’s fairly tasteless, I’ll put gravy on it.”
	Meal includes sauce: Decision to add SS is affected by the presence or absence of existing SS	P16: “We buy a potato salad coleslaw and that’s already got its sauce on it. So, I wouldn’t put any more on it.”
Variation	Dependent on dining environment: SS usage is based on dining location (home/restaurant)	P5: “I do enjoy sauces on food. I just don’t do it myself at home.” P6: “The only time I would have a sauce with meat, would be if I was out to dinner. I wouldn’t have it at home.”
	Seasonally dependent: SS ingredients are dependent on the season	P4: “At Christmas time, when we have turkey, I always have cranberry sauce.” P7: “I make gravy with liver. I have granules for that, but that only comes out in the winter.”
	Location/Resource dependent: SS ingredients are reliant on availability, often dependent on the location or climate	P14: “In Yorkshire, there’s Henderson’s relish, not as hot, but we have to go up to Yorkshire to get it.” P18: “If I’ve got mint in the garden, I’ll make the mint sauce. If you know, it’s the middle of winter and I don’t want to go out and pick the mint, I’ll make the sauce from a bought mint jar.”
	Meal-specific choices: The use of SS ingredients is specific to each meal due to perceived appropriate sauce and meal pairings	P2: “(I use) gravy if I’m having a roast.” P3: “Mustard with pork, ketchup maybe with fish and chips.”
Individual Differences	Personal Preferences: Preferences and perceptions that may impact SS usage, including perceptions of overuse or excessive use	P4: “I can’t eat English mustard; I find it far too strong and powerful.” P20: “Ketchup I can’t stand, brown sauce I will use.”
	Personal Experiences: Personal experiences, such as habits, upbringing and past experiences, specific to the individual, that influence SS usage	P6: “I always season everything.” P14: “I’ve watched the way my mother used to conjure meals out of not a lot of money, and I think that is a tendency in my family.” P19: “People have different opinions (on sauces) based on the way they’ve been brought up. It is part of our heritage in terms of going back centuries, there were always these kinds of accompaniments.”
	Ethical choices: Food choices driven by ethical or religious viewpoints	P16: “We only use onion gravy, because our daughter’s vegetarian she doesn’t like beef gravy and I always use onion gravy.”
	Health Beliefs: Health beliefs that may influence SS usage, including belief in one’s own health/diet and that SS are not necessary, health-related connotations that are attached to certain foods/ingredients, and an influence of health conditions	P4: “I love garlic because garlic is very good for you. It’s got a lot of good properties.” P9: “I suffer from a little bit of low blood pressure, so I need salt.” P20: “It’s a good way to go on a diet, forget the sauces and seasonings, I won’t eat as much.”
	Personal Circumstances: Aspects of personal circumstance that may influence SS usage, including living status, mobility, time, effort, cooking ability and finances	P1: “(I) haven’t got a lot of money now.” P2: “When my husband was alive it was different, but now I’m on my own I don’t do things like that (adding herbs).” P2: “Standing up, I can’t stand up very well and that is one reason I don’t try recipes requiring sauces.” P5 “I’m not very good (at cooking), I just like nice easy meals.”
	Age-related concerns: Physiological concerns that occur with age and may affect SS usage, such as deteriorations in taste abilities and digestive problems	P1: “I don’t like spicy sauces. They give me a bit of heartburn and indigestion. So, I don’t eat them anymore.” P9: “As you get older you lose a little bit of your taste buds..., so therefore, to increase the taste of food, something artificial has got to be added to it, like a sauce.”

SS: sauces and seasonings.

**Table 4:** Mean, SD score from −2 to +2 for all attitudinal statements in the questionnaire on future sauce and seasoning use, in the sample as a whole (*N* = 22). Statistics (t values) demonstrate a difference from 0 (no opinion).

	Mean	SD	t(21)	*p*
Using SS in future would add more flavour/taste to my foods	0.5	1.3	1.78	0.09
Using SS in future would make my food more pleasant	0.2	1.4	0.62	0.54
**Using SS in future would cause me discomfort/pain**	**−1.1**	**0.9**	**−5.48**	**<0.01**
Using SS in future would make my food easier to eat/encourage me to eat more food	−0.3	0.9	−1.62	0.12
Using SS in future would benefit my health/encourage me to eat more healthy food	−0.3	1.1	−1.45	0.16
I intend to/it is likely that I will use more SS in future	−0.1	1.1	−0.57	0.58
**I am confident/in control/would have no trouble using more SS in future**	**1.2**	**0.6**	**9.54**	**<0.01**
**Having thought about SS, I am concerned that my diet is unhealthy/I should be thinking more about my diet/my health, and thinking about my diet/health has upset me**	**−1.2**	**0.8**	**−7.28**	**<0.01**

SS: sauces and seasonings. Significance set at *p* ≤ 0.01, significant effects are highlighted in bold.

## Data Availability

Data are available from the corresponding author on reasonable request.

## Supplementary Materials

The following are available online at https://www.mdpi.com/article/10.3390/foods10112828/s1, Table S1: (Table 2: extended): extended: Mean, SD, minimum and maximum score from −2 to +2 in agreement with all attitudinal statements in the questionnaire on sauce and seasoning use and attitudes, in the sample as a whole (*N* = 22), and in high (*N* = 7), moderate (*N* = 9) and low (*N* = 6) users. Statistics (t values) demonstrate a difference from 0 in the sample as a whole, and (F values) demonstrate a difference between low, moderate and high users of added flavours. Table S2: (Table 3: extended): extended: Themes table from the qualitative analysis of the interviews, demonstrating themes, subthemes and example quotes.
