# Supplementary material for: Adding Flavours: Use of and Attitudes towards Sauces and Seasonings in a Sample of Community-Dwelling UK Older Adults
Source: Foods. 2021 Nov 17;10(11):2828. doi: 10.3390/foods10112828 (PMC8619839; doi:10.3390/foods10112828)
Supplement: Supplementary file 1 [file foods-10-02828-s001.zip › foods-1472999-supplementary.pdf]

ADDING FLAVOURS: USE OF AND ATTITUDES TOWARDS SAUCES AND SEASONINGS IN COMMUNITY-DWELLING UK OLDER ADULTS

Annie Thomas, Charlotte Boobyer, Zara Borgonha, Emmy van den Heuvel, Katherine M Appleton
Research Centre for Behaviour Change, Department of Psychology, Faculty of Science and Technology, Bournemouth University, UK.

SUPPLEMENTARY MATERIALS

Methods: Overview of research provided to participants during the interview.

ADD SAUCES AND SEASONINGS TO INCREASE PROTEIN INTAKES IN OLDER ADULTS

Professor Katherine Appleton, Bournemouth University

Many older adults eat only low amounts of good quality high-protein foods, including meat, fish, eggs and dairy products, but the low consumption of dietary protein can have many negative impacts on health, from reduced mobility and independence, to increased risk of fall, fractures and long hospital stays. To try and increase the consumption of high protein foods, we have been looking at the possible impacts of adding taste, familiarity and enjoyment to foods in the form of sauces and seasonings. We have now completed three studies, where older adults came to the laboratory for us on two occasions, and on one occasion consumed a hot chicken meal with sauce or seasoning, and on another occasion consumed the same meal with no sauce. In all three studies, participants consumed more chicken, and so more good quality protein, from the meal with sauce or seasonings compared to the plain meals. In the final study, the majority, but not all individuals were aided by the sauce, and effects at lunch were maintained over the following meal, so those who did benefit from the sauce showed higher protein intakes over both meals. Our research demonstrates that adding sauces and seasonings to meals can increase the consumption of high-protein foods for older adults, and that these effects are sustained over the next meal.

References

Research conducted by Prof. Katherine Appleton, Bournemouth University

- Appleton KM (2009) Increases in energy, protein and fat intake following the addition of sauce to an older person's meal, Appetite, 52, 161-165
- Best RL, Appleton KM. (2011) Comparable increases in energy, protein and fat intakes following the addition of seasonings and sauces to an older person's meal. Appetite, 56, 179-182
- Appleton KM (2018) Limited compensation at the following meal for protein and energy intake at a lunch meal in healthy free-living older adults, Clinical Nutrition, 37, 970-977.

Table S1: (Table 2: extended): Mean, SD, minimum and maximum score from -2 to +2 in agreement with all attitudinal statements in the questionnaire on sauce and seasoning use and attitudes, in the sample as a whole (N=22), and in high (N=7), moderate (N=9) and low (N=6) users. Statistics (t values) demonstrate a difference from 0 in the sample as a whole, and (F values) demonstrate a difference between low, moderate and high users of added flavours.

	Sample as a whole				Sample divided as low, moderate and high users							
	Whole sample (N=22)				Low (N=6)		Moderate (N=9)		High (N=7)			
	Mean	SD	t(21)	P	Mean	SD	Mean	SD	Mean	SD	F(2,21)	p
**SS add flavour to foods / make foods more tasty**	**1.0**	**0.8**	**6.08**	**<.01**	0.8	0.7	1.1	0.9	1.1	0.7	0.43	0.65
**SS make foods more pleasant / likeable**	**0.6**	**0.9**	**2.95**	**<.01**	-0.1	0.8	0.6	0.8	1.1	0.7	3.76	0.04
**SS disagree with me / cause me pain**	**-1.1**	**0.7**	**7.78**	**<.01**	-1.0	0.8	-0.8	0.6	-1.6	0.3	2.97	0.08
I am used to using SS	0.2	1.3	0.70	.49	-0.3	1.5	0.2	1.3	0.8	0.8	1.08	0.36
SS encourage me to eat more food / make food easier to eat	-0.4	0.9	1.84	.08	-0.8	0.8	0.2	1.0	-0.6	0.7	3.05	0.07
**SS encourage me to eat more healthy food / contribute to a healthy diet**	**-0.6**	**0.9**	**2.95**	**<.01**	-1.1	0.9	-0.2	0.8	-0.6	0.9	1.75	0.20
**I prefer to add my own SS as sometimes there can be too much**	**1**	**1.0**	**4.82**	**<.01**	1.0	1.5	1.1	0.6	1.2	1.1	0.05	0.95
Dishes without SS do not taste pleasant	0	1.3	0	.99	-0.7	1.4	0.1	0.9	0.6	1.7	1.42	0.27
**It is important to me that the foods I eat are pleasant / enjoyable**	**1.5**	**0.5**	**13.10**	**<.01**	1.2	0.5	1.8	0.4	1.5	0.6	2.62	0.10
**It is important to me that the foods I eat are tasty**	**1.4**	**0.5**	**13.58**	**<.01**	1.2	0.4	1.8	0.4	1.6	0.5	3.22	0.07
It is important to me that the foods I eat are familiar to me	-0.4	1.2	1.51	.02	0.5	1.0	-0.4	1.0	-1.4	0.9	4.96	0.02
**It is important to me that the foods I eat are healthy / I try to have a healthy diet**	**1.4**	**0.7**	**9.66**	**<.01**	1.4	0.7	1.6	0.5	1.2	0.9	0.62	0.55
**My health is important to me**	**1.6**	**0.5**	**15.39**	**<.01**	2.0	0	1.8	0.4	1.6	0.9	0.79	0.47
It is important to me that other people think I have / would not criticise my diet	-0.3	1.0	1.30	.21	-0.3	1.1	-0.2	1.1	-0.4	1.0	0.04	0.97
It is important to me that other people think I am healthy / not unhealthy	0	0.9	0.23	.82	-0.1	1.2	0.3	0.9	-0.3	0.8	0.72	0.50

SS: sauces and seasonings. Significance set at p ≤ 0.01, significant effects are highlighted.

Table S2: (Table 3: extended): Themes table from the qualitative analysis of the interviews, demonstrating themes, subthemes and example quotes

Theme	Subtheme	Example Quotes
Meal Enhancement	Improves taste: Added SS improve the taste of the meal.	P1: "It's a hearty meal. (..) It's just the herbs that make the taste."<br>P3: "I may put some herbs in it. I may add a sauce, to make them (foods) taste nicer."<br>P4: "I have my cup of soup and I put onion powder, garlic powder, black pepper and chilli .., it makes the soup taste quite nice."<br>P9: "I add on Lea and Perrins (Worcestershire sauce) to make it a little bit stronger in taste."<br>P16: "Salmon hasn't got much of a taste for me, it hasn't, so really that deserves a sauce."
	Enhances smell: Added SS improve the overall smell of foods.	P1: "Rosemary it smells very good."
	Increases flavours: There are more flavours in the dish when SS are added	P3: "Add herbs, which do add a lot of flavour."<br>P3: "If you add lemon to fish, it enhances the flavour. If you add herbs to eggs, an omelette, it adds more flavours."<br>P4: "Add herbs and spices and stuff like that. That usually helps to improve flavours."<br>P4: "People like to have meat and fish like that with a sauce on. Gives it a bit of added flavour."<br>P10: "Salt does add flavour."
	Changes in the composition / texture of foods: Added SS change the overall texture of the meal	P7: "I've discovered chilli sauce and I put it on chips, as it seems to counteract any grease."<br>P9: "I make my own onion gravy ... I add a bit of flour to give it a bit more texture."
	Aiding digestion / ability to eat: Added sauces can improve digestion, often due to texture and lubrication	P1: "Adding sauces to food helps it go down more easily."<br>P17: "It's (sauce) a lubricant though, which is an aid to swallowing."<br>P17: "Teeth make a difference as well. My teeth are still good. With older people, not having proper teeth is huge. Particularly if you're going out with people, you're more restricted on what you can choose."
	Cooking enjoyment: Enjoyment in cooking with using additional ingredients	P20: "I have parsley, sorrel, sage, rosemary, rocket, bay tree, just about everything you can mention. I really like cooking with flavourings."

Theme	Subtheme	Example Quotes
The Product Itself	Product ingredients: Importance of the specific ingredients, often with a focus on natural ingredients	P1: “I add a little bit of tomato ketchup and it’s naturally based with peppers.”<br>P9: “Her own sauces are a little bit fresher.”<br>P13: “I will make my own sauce to go with the meal rather than buy a bottle. Purely because what’s in the bottle, you don’t know what’s in it.”<br>P21: “The first thing I’d do is look at the ingredients ... What I don’t like is you see they’ve got added sugar ... If they’ve got added sugar I wouldn’t buy them.”
	Product reputation: The reputation of a product influences SS usage	P12: “I do tend to buy those off the shelf. But again, certain brands that I know and that I trust.”
	Shelf life / Longevity: Product recommended shelf life influences SS usage	P1: “A drum of salt can last me five plus years.”<br>P2: “You can’t just buy a jar because it goes off, so I don’t have it in the house.”
	Ingredient wastage: Likely product waste can influence SS usage	P2: “I did go to (Cash & Carry) and you can buy what they use in hotels, so I buy the sachets of salt / pepper.”<br>P6: “You buy a whole bottle of, say fish sauce, you use a tablespoon then the bottle gets thrown away.”<br>P5: “I don’t have a lot of wastage, so I wouldn’t buy in things that I won’t use.”<br>P17: “What might put me off, if I had to go and buy 10 pots of particular spices that I wouldn’t be using again, and they’d be sitting around in your cupboard.”
Characteristics of the Meal	Preference for natural flavourings / taste: A preference for natural tastes that can affect SS usage	P4: “I like to taste my food and know what I’m eating.”<br>P6: “If the food tastes fine, I don’t add anything to it.”<br>P6: “If I wanted a cheese sauce I would make a cheese sauce. I wouldn’t dream of buying it in a packet, because it doesn’t remotely resemble a proper cheese sauce.”<br>P7: “I prefer to taste my food natural, I don’t want the enhancement of something else to interfere with it.”<br>P10: “It would add this very artificial flavour. All these salad dressings that you get they affect the flavour of the whole thing.”<br>P13: “If you like chicken why spoil it?”

Theme	Subtheme	Example Quotes
Characteristics of the Meal continued	Quality ingredients: Perceived quality of the meal ingredients influences SS usage	P1: "If I buy this good quality meat ..., I just add a little bit of seasoning, a very low amount."<br>P7: "If the meat is decent then you have the flavour in it, so you shouldn't need to mess it about with sauces."<br>P12: "If the meats fairly tasteless, then I'll put gravy on it."<br>P12: "If I'm eating a steak or something, then I would tend not to use them (sauces or seasonings). I tend to want to enjoy the juices within the meat that I'm eating."<br>P19: "It (sauce) would alter the flavour of the meat and fish I think. If it wasn't a particularly good cut of meat, I would put it in a casserole and add garlic and herbs."
	Meal includes sauce: Decision to add SS is affected by the presence or absence of existing SS	P16: "We buy a potato salad coleslaw and that's already got it its sauce on it. So I wouldn't put any more on it."
Variation	Dependent on dining environment: SS usage is based on dining location (home/restaurant)	P5: "I do enjoy sauces on food. I just don't do it myself at home."<br>P6: "The only time I would have a sauce with meat, would be if I was out to dinner. I wouldn't have it at home."
	Seasonally dependent: SS ingredients are dependent on the season	P4: "At Christmas time, when we have turkey, I always have cranberry sauce."<br>P7: "I make gravy with liver. I have granules for that, but that only comes out in the winter."<br>P7: "At Christmas, I use bread sauce and I make that myself from milk and onions."<br>P10: "I would normally use them (stock cubes) more in the winter."<br>P17: "If the mint was in season, I'd use it, otherwise I'd have mint jelly."<br>P20: "We use spices when we make red cabbage, but we do that in the winter."
	Location / Resource dependent: SS ingredients are reliant on availability, often dependent on the location or climate	P10: "I lived in Portugal for a long, long time and I put olive oil on veggies, potatoes, everything."<br>P14: "In Yorkshire, there's Henderson's relish, not as hot, but we have to go up to Yorkshire to get it. Get it by the case."<br>P17: "I'd add chopped parsley, mainly herbs. I had a herb garden in my previous house and haven't got one here."<br>P18: "If I've got mint in the garden, I'll make the mint sauce. If you know, it's the middle of winter and I don't want to go out and pick the mint, I'll make the sauce from a bought mint jar."<br>P20: "We have a place in France and whenever we go there I'll bring back a box of wine and I always keep one in the fridge so it's easy to access when I'm cooking."<br>P21: "I grow the herbs, I just go out and take whatever takes my fancy."<br>P21: "I grow my own parsley, so if I was going to make parsley sauce I'd make it."

Theme	Subtheme	Example Quotes
Variation continued	Meal specific choices: The use of SS ingredients is specific to each meal due to perceived appropriate sauce and meal pairings	P2: “(I use) gravy if I’m having a roast.”
		P3: “Mustard with pork, ketchup maybe with fish and chips.”
		P6: “I would use fresh herbs and spices, if I was making a curry obviously.”
		P8: “I like tartar sauce with fish, I usually have mint sauce with lamb.”
		P8: “With a roast dinner I wouldn’t want it without any sort of gravy.”
		P9: “I made myself a chicken curry, in that curry was a mixture of spices, turmeric, cumin.”
Individual Differences	Personal Preferences: Preferences and perceptions that may impact SS usage, including perceptions of overuse or excessive use	P1: “I prefer cheese to top my vegetables, it’s almost on the same level as sauces and seasonings."
		P4: “I can’t eat English mustard, I find it far too strong and powerful.”
		P10: “I think the more one drenches foods in all these flavours and sauces, the more it kills ones taste buds and it becomes bland.”
		P17: “It doesn’t take a second. And I prefer it. What I do, if I’m making a sauce, I’ll make it in a large quantity and I’ll freeze that.”
		P18: “Ready meals are so easy to get now, but we’ve had to write to (Food manufacturer) and other food manufacturers so often because the readymade meals are so salty that we can’t have them.”
		P19: “I very rarely buy the packet sauces. I just don’t like the taste.”
		P20: “Ketchup I can’t stand, brown sauce I will use.”
	Personal Experiences: Personal experiences, such as habits, upbringing and past experiences, specific to the individual, that influence SS usage	P1: “It’s a little bit hereditary, you know, what I had at home.”
		P1: “I learned from my mother, once you have a soup, you can take a bit of the soup and take a bit of cornflower (..) and make a sauce.”
		P6: “I always season everything.”
		P6: “I always use salt and pepper.”
		P7: “I’m so used to cooking what I normally cook that I don’t experiment.”
		P8: “I use a basic mixture of spices, and some of the little packs of seasoning.”
		P13: “I like particularly Moroccan and Middle Eastern and South American (spices). South American flavours are just starting to take off now, particularly recipes that come out of Paraguay are very nice.”
		P14: “I’ve watched the way my mother used to conjure meals out of not a lot of money and I think that is a tendency in my family.”
		P18: “I’ve worked in the Middle East and Arabic food is often ruined by sauces and spices, which take away the taste of the basic food you’re eating.”
		P18: “I went to grammar school for a while, and we got off the bus next to the HP sauce factory, and the fumes were so horrific first thing in the morning that I could never touch anything like that.”

Theme	Subtheme	Example Quotes
Individual Differences continued	Personal Experiences: Personal experiences, such as habits, upbringing and past experiences, specific to the individual, that influence SS usage continued	P19: “People have different opinions (on sauces) based on the way they’ve been brought up. It is part of our heritage in terms of going back centuries, there were always these kinds of accompaniments.”<br>P20: “If I’m making a curry I’ll use lots of spices. I have an Indian friend who sends me some.”
	Ethical choices: Food choices driven by ethical or religious viewpoints	P8: “There are three vegetarians and four meat eaters, so we have to do a lot of bits and pieces … we have gravy with the chicken (dishes).”<br>P16: “We only use onion gravy, because our daughter’s vegetarian she doesn’t like beef gravy and I always use onion gravy.”
	Health Beliefs: Health Beliefs that may influence SS usage, including belief in one’s own health / diet and that SS aren’t necessary, health-related connotations that are attached to certain foods / ingredients, and an influence of health conditions	P1: “I use rice bran oil, it’s 100% non-cholesterol.”<br>P1: “I use Marigold bouillon, which is natural … It’s in the genes sometimes … allergies, but to try and minimize the chances of allergies I eat this.”<br>P4: “I love garlic because garlic is very good for you. It’s got a lot of good properties.”<br>P7: “(Sauces) wouldn’t encourage me to eat more, because I have a basic diet which I think is a balanced one.”<br>P10: “Maybe perhaps (there are health benefits) when I make my own, because there’s less sugar in it”<br>P9: “I’ll add some butter or (low fat spread) when I’m cooking, because I’ve told you I have this heart condition, something which is lower in fat like (low fat spread).”<br>P9: “I suffer from a little bit of low blood pressure, so I need salt.”<br>P10: “They’ve (sauces) have got too much sugar in and probably too much salt as well.”<br>P13: “I make my own garlic butter and you can get the Lurpak lightest spreadable and its about 4 calories.”<br>P13: “The mustard that you buy in a regular jar has too much fat in it.”<br>P12: “There’s too many calories for us in the sauces.”<br>P16: “That’s why I’m so slim because … I don’t have all these bits and pieces.”<br>P16: “I would always think that they’re (sauces) fattening and as I can’t lose weight anyway I don’t want to put any on.”<br>P20: “I’m trying to lose weight at the moment, so today I had a jacket potato and I just put baked beans on it, instead of using butter as a topping.”<br>P20: “It’s a good way to go on a diet, forget the sauces and seasonings, I won’t eat as much.”

Theme	Subtheme	Example Quotes
Individual Differences continued	Personal Circumstances: Aspects of personal circumstance that may influence SS usage, including living status, mobility, time, effort, cooking ability and finances	P1: “(I) haven’t got a lot of money now.”<br>P1: “If I see basil or rosemary, things that I know I use in a recipe, I buy it fresh and I freeze it.”<br>P2: “When my husband was alive it was different, but now I’m on my own I don’t do things like that (adding herbs).”<br>P2: “Not just for me. When you’re on your own, you don’t do that (adding stock cubes).”<br>P2: “To be honest with you as I live on my own, I don’t do much cooking, so I won’t use them (sauces).”<br>P2: “Standing up, I can’t stand up very well. And that is one reason I don’t try recipes requiring sauces.”<br>P3: “Lack of time. I just don’t have time to try to recipes with sauces.”<br>P4: “I don’t cook much now, because being on my own, I just live on sandwiches and microwave meals.”<br>P4: “It’s (cooking) not worth putting the effort in.”<br>P5 “I’ve been on my own a long time now, so usually it’s something quite plain that I eat.”<br>P5 “I’m not very good (at cooking), I just like nice easy meals.”<br>P9: “I can’t be bothered. Lazy…, it is…, it’s pure laziness.”<br>P17: “And shopping, access to the shops. Because if you can’t drive, you see people arriving at the shops in a taxi.”<br>P17: “I haven’t made mango chutney, because mango is quite expensive and you don’t use that much of it.”
	Age-related concerns: Physiological concerns that occur with age and may affect SS usage, such as deteriorations in taste abilities and digestive problems	P1: “I don’t like spicy sauces. They give me a bit of heartburn and indigestion. So, I don’t eat them anymore.”<br>P9: “As you get older you lose a little bit of your taste buds ..., so therefore to increase the taste of food something artificial has got to be added to it, like a sauce.”<br>P13: “Because my taste buds are a bit weird, I’d like to have cayenne pepper which is a bit sharper, bit stronger than ground pepper.”<br>P14: “The other thing is fat, it gives me indigestion now that I’m older.”<br>P17: “Your digestion isn’t as efficient. You’re more likely to have things like irritable bowel syndrome, which is exacerbated by strong spices.”

SS: sauces and seasonings.
